# Influence of Ligand Functionalization on the Synthesis of Metallic-Decorated Magnetic Nanoparticles for Antibacterial Treatment

**DOI:** 10.1021/acsanm.6c02039

**Published:** 2026-06-24

**Authors:** Allison L. Stadick, Laura Scala, Juan L. Vivero-Escoto

**Affiliations:** 14727 University of North Carolina at Charlotte, 9201 University City Blvd, Charlotte, North Carolina 28223, United States

**Keywords:** nanoparticles, antibacterial, iron oxide, metallic, hard/soft acid/base theory

## Abstract

The inherent magnetism of iron oxide nanoparticles (IONPs) provides appealing benefits for antibacterial treatment, as IONPs can be readily guided, concentrated, and removed from a specific site. Additionally, gold and silver demonstrate antibacterial properties that effectively inhibit bacterial growth. By combining one of these antibacterial metals and the IONP, a dual-purpose metallic nanoparticle treatment can thus be constructed. In our study, we developed silica-coated IONPs to facilitate the binding and decoration with either gold (Au) or silver (Ag). Therefore, the focus of this research is to apply the hard/soft acid/base (HSAB) theory by investigating the affinity of Au or Ag after encapsulating the IONPs with one of three silane capping agents, providing either an amine (AP-SIONP), hydroxide (T-SIONP), or thiol (MP-SIONP). With the use of inductively coupled plasma optical emission spectroscopy (ICP-OES), the amount of metal decorated on the antibacterial metallic SIONPs was compared. We demonstrated that although both Au and Ag had an affinity for all three ligands, Au (79 ± 18 and 23 ± 0 μg/L) and Ag (72 ± 36 and 160 ± 23 μg/L) had a higher affinity for amines and thiols, respectively. Finally, the optimal Au and Ag SIONPs were applied to a Gram-negative (*Escherichia coli*) and Gram-positive (*Staphylococcus aureus*) bacterium to investigate their antibacterial and capturing potential. Our findings indicate that AgMP-SIONPs demonstrated superior antibacterial potential by providing inhibitory concentrations at 62.5 and 500 μg/mL for *E. coli* and *S. aureus*, respectively. Moreover, AgMP-SIONPs provided a minimum bacterial concentration (MBC) at 62.5 μg/L but did not reach MBC for the Gram-positive bacterium. Overall, this study provides the protocol for an optimal antibacterial metallic SIONP through the application of the HSAB theory and demonstrated the promise of silver for its antibacterial potential and SIONP ability to further capture bacteria, all of which opens a promising research exploration.

## Graphical abstract

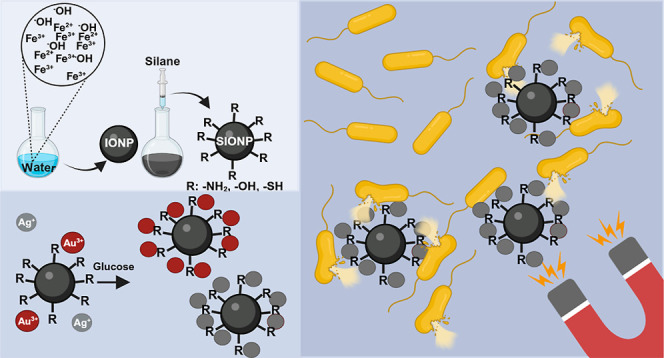

## 1 Introduction

Pathogenic bacteria is one of the leading causes of deaths worldwide, an issue that is fueled by the increase in bacterial resistance to antibiotics and poor accessibility to medical care in developing countries.^1,2^ Pathogenic bacteria can easily spread from an infected individual or surface to another individual, causing further infection and potentially severe harm.^3^ Although antibiotics have been developed to treat these bacterial infections in the past, a concerning rise in bacterial resistance to these antibiotics has created an urgent demand for new methods to treat and prevent infections.^4^ Inorganic nanoparticles (NPs) hold promising potential in the field of infectious diseases because they provide a broad spectrum of antibacterial properties due to their affinity for many of the biomolecules found within the bacteria; these interactions cause irreversible damage, increasing the treatment potency.^5–7^ Moreover, inorganic NPs present durability and stability contributing to a greater shelf life and more reliable application to harsher microenvironments. Overall, inorganic NPs are an ideal and novel candidate for future antibacterial treatments.^5^ Three inorganic NPs that are gaining recognition for the treatment of pathogenic bacteria are iron oxide (IO), silver (Ag), and gold (Au). One pertinent property of iron oxide nanoparticles (IONPs) is their magnetism: using an external magnetic field, the IONPs can be concentrated at a site of infection or guided through complex biofilms, then finally removed.^6,8^ Additionally, Ag and Au nanomaterials are of interest due to their antibacterial properties and ease of synthesis, making them ideal for manufacturing.^9–13^ Furthermore, metallic NPs provide additional benefits due to their plasmonic properties which can aid in biosensing,^14–16^ and photothermal^17^ or photodynamic therapy.^18^

Although IONPs provide great benefits due to the magnetic features, precursor chemical availability, and ease of synthesis, they can quickly oxidize under aqueous conditions, reducing their magnetization and potentially contributing to increased cytotoxicity.^19,20^ To encapsulate and prevent oxidation of IONPs, the sol–gel method with silane precursors has been widely used. Furthermore, silica can provide improved hydrophilicity, bioavailability, and lowered cytotoxicity.^21–23^ Several synthetic methods have been reported with the use of silane agents; however, many incorporate the use of organic solvents, toxic reagents, high temperature, high pressure,^24–26^ and intermittent washing steps.^27,28^ All of these methods could introduce hazardous and complex synthesis or introduce oxidation and reduce magnetism of the IONPs, respectively. To our knowledge, none of these methods provide a one-pot synthetic approach in aqueous conditions to functionalize IONPs. In this work, using a one-pot method, IONPs are synthesized with three different silane capping agents that provide either an amine (AP-SIONP), hydroxide (T-SIONP), or thiol (MP-SIONP) (Figure 1). These SIONPs were characterized to confirm structural integrity with powdered X-ray diffractions (PXRD) and Fourier transform infrared spectroscopy (FTIR). The dispersions of the IONPs and SIONPs were analyzed with dynamic light scattering (DLS). The three functional groups (−OH, –NH_2_, and –SH) were confirmed with zeta potential analysis and colorimetric assays. Moreover, we further combined these IONPs with an antibacterial metal (Ag or Au) to synthesize NPs under aqueous conditions and with the use of glucose as a reducing agent (Figure 1). The successful synthesis of these metallic NPs was confirmed by UV–vis, transmission electron microscopy (TEM), inductively coupled plasma-optical emission spectroscopy (ICP-OES), and dark field microscopy/scattering (DFM/S). We optimized the one-pot synthesis of metallic SIONPs by applying the hard/soft acid/base (HSAB) theory. HSAB can predict the metal–ligand interaction between the specific ligands (−OH, –NH_2_, and –SH) and metal precursors (Ag^+^ and Au^3+^).^29,30^

**Figure 1:**
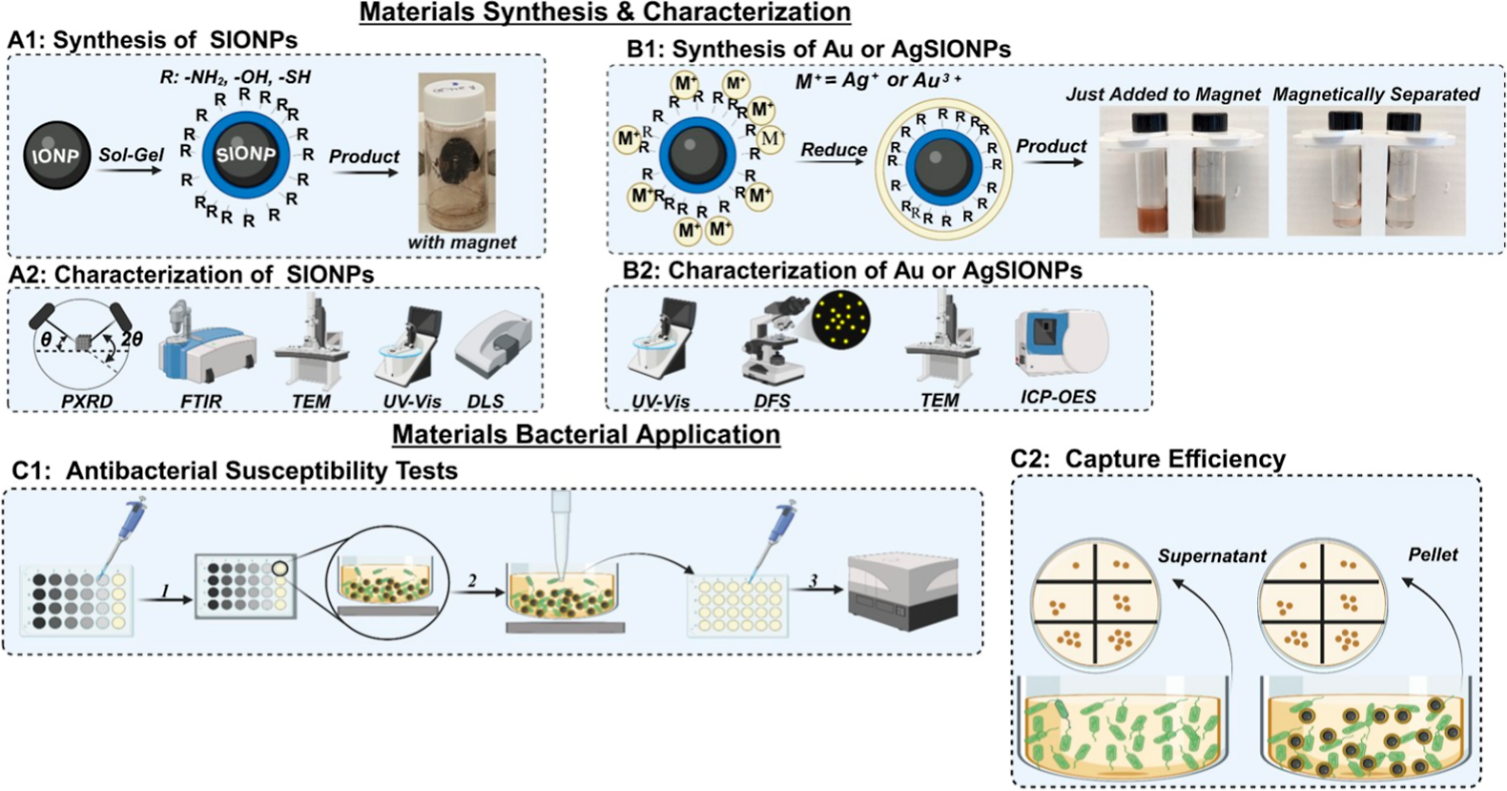
A flowchart for the synthesis, characterization, and application of metallic-coated SIONPs. The first step (A1) involves the one-pot synthesis of IONPs and capping with a silica precursor to afford SIONPs. The SIONPs crystallinity, structure, colloidal stability, and ligand are analyzed (A2). The second step involves the coating of the SIONPs with either Au or Ag by metal ligand interaction and reduction (B1). The samples are then analyzed with ICP-OES to quantify the amount of antibacterial metal, TEM to analyze the morphology, and DFM to analyze the homogeneity (B2). Finally, the optimal antibacterial SIONPs are applied to *E. coli* (K12 strain) (C).

Lastly, the antibacterial properties and capture efficiencies of the metallic SIONPs were investigated against *Escherichia coli* (K12 strain) and *Staphylococcus aureus* (Xen 40) bacteria (Figure 1). We envision that this novel synthetic approach that takes advantage of the knowledge of HSAB theory to develop optimized metallic NPs can be used to fabricate efficient nanomaterials to eliminate bacteria.

## 2 Experimental Section

### 2.1 Materials and Methods

The following chemicals were purchased from the respective suppliers and used without modifications. Hydrochloric acid (HCl) (Macron chemicals), nitric acid (HNO_3_) (Macron chemicals), Barnstead Nanopure Water (18 MΩ), ferric chloride (FeCl_3_) (Sigma-Aldrich, reagent grade, 97%), ferrous chloride tetrahydrate (FeCl_2_·4H_2_O) (fisher science education, reagent grade), sodium hydroxide (NaOH) (fisher chemical, certified ACS), tetraethyl orthosilicate (TEOS) (Sigma-Aldrich, ≥99.0%), 3-mercaptopropyltriethoxysilane (MPTES) (Gelest, 95%), (3-aminopropyl)­triethoxysilane (APTES) (Alfa Aesar, 98%), ninhydrin (ACS reagent), dithiothreitol (DTT) (Cleland’s reagent, molecular biology grade), anhydrous d­(+)-Glucose (ACS reagent), silver nitrate (AgNO_3_) (ACS reagent, 99+%), *N*,*N*-diisopropylethylamine (DIPEA) (≥99%, *Reagent Plus*), gold­(III) chloride trihydrate (HAuCl_4_·3H_2_O) (ACS reagent grade, ≥49% Au basis), Dulbecco’s phosphate buffered saline (DPBS), 190 proof pure ethanol (KOPTEC), Luria–Bertani Broth (Miller, Sigma-Aldrich), Brain Heart Infusion Broth (ThermoFisher Scientific), Agar (bacteriological, VWR), and Mueller Hinton Broth (VWR).

### 2.2 Synthesis of Nanoparticles

All glassware used for the synthesis and characterization of NPs was cleaned with aqua regia (3 parts in volume of hydrochloric acid and 1 part in volume of nitric acid), followed by soaking in a base bath (hydroxide and isopropyl alcohol) prior to use.

#### 2.2.1 One-Pot Synthesis of Silica-Capped IONPs

To synthesize IONPs, FeCl_3_ (270.3 mg) and FeCl_2_·4H_2_O (167.8 mg) were dissolved in 26 mL nanopure water to make 0.1 M iron solution (2:1 Fe^3+^ to Fe^2+^ molar ratio). The water was initially pH adjusted to pH = 1.8 with 2.0 M HCl. The iron solution was placed in an oil bath set at 60 °C and purged with nitrogen gas for 10 min. Afterward, under the continuous flow of nitrogen, 1.0 M NaOH (12.5 mL) was added dropwise over 10 min and the rpm was adjusted based on viscosity (600–1000 rpm) to ensure a defined vortex. During the addition of the NaOH, the reaction turned from a yellow, to a rust red, and finally a black color. This black color indicates the formation of magnetite (IONPs). Afterward, the solution was stirred for 30 min and then 1 mL of 1.14 M TEOS (containing 60% ethanol) was added dropwise over 5 min and the reaction was stirred for 2 h. For TEOS-SIONPs (T-SIONPs), this step was elongated to 24 h and the T-SIONPs were magnetically separated and washed with water and ethanol separately. Finally, APTES (5.7 mL) or MPTES (0.27 mL) were added dropwise over 15 and 5 min, respectively. This solution was left to mix for another 24 h at 60 °C under the continuous flow of nitrogen gas. The SIONPs were magnetically separated and washed with water and ethanol separately. Finally, the NPs were lyophilized and stored in a desiccator. IONPs were synthesized without silica coating as a control for chemical stability and crystallization comparison.

#### 2.2.2 Gold and Silver Decoration of Silica-Capped IONPs

T-SIONPs and AP-SIONPs (5 mg) were separately dispersed in water (concentration of 0.1 mg/mL) and sonicated for 30 min or until fully dispersed. The SIONPs were intermittently vortexed to aid in dispersion. Then 154 μL of aqueous 1.8 M HAuCl_4_·3H_2_O or same amount of AgNO_3_ were added to the SIONP solution. The SIONP-metal solution was mixed for 45 min at room temperature followed by placing the reaction vessel in an ice bath and adding DIPEA to obtain pH ∼ 6. After 15 min, 5 mL of aqueous 0.2 M glucose was added dropwise. After 48 h, the solution was washed 3 times with water or until the supernatant was clear. AuAP-SIONPs, AgAP-SIONPs, AuT-SIONPs, or AgT-SIONPs was then redispersed in water and stored in the dark at 4 ° C.

A similar protocol was used for the MP-SIONPs; however, the MP-SIONPs had to react with DTT first to reduce the disulfide bonds formed during MP-SIONP synthesis. Briefly, an aqueous solution of DTT (75 mL, 10 mM) was adjusted with DIPEA to achieve pH 8.0. Then, IONPs (25 mg/mL) were added and left to stir for 6 h. The MP-SIONPs were magnetically separated and washed with water three times before being reacted with Au or Ag metals to afford AuMP-SIONPs or AgMP-SIONPs, respectively. An experiment was performed to determine the optimal reaction times for treating MP-SIONPs with DTT at 0, 2, 6, 18, and 24 h, and a colorimetric assay described in Section 2.3.2 was used to calculate the quantity of thiols per mg of MP-SIONPs.

### 2.3 Characterization

#### 2.3.1 Fabrication of Magnetite and NPs

The lyophilized samples of the SIONPs and IONPs were characterized by a Goniometer Oxford Diffraction KM4 XCALIBUR2 (295.4 K, shield flow of 4.0 L/min, sample flow of 6.0 L/min, Cu X-ray) to analyze the impact the silica coating has on the structural integrity of the magnetite crystalline phase. Images of the SIONPs were obtained using a JEOL 2100 LaB6 Transmission Electron Microscope (TEM), 200 kV, and complementary AZtec electron-dispersive spectrometry (EDS) (Aztec 6.0 software, input count rate <30,000, output count rate <30,000, dead time <10%) for that solution containing 10 μg/mL sample in methanol, with 5 μL dropped onto a 200 lacey mesh copper grid.

#### 2.3.2 Silica Coating and Ligand Functionalization

The dried samples were analyzed with a PerkinElmer FT-IR Spectrometer Spectrum 3. SIONPs were dispersed in water via sonication (30 min) and 0.1X DPBS buffer/water solution at 0.01 mg/mL and analyzed with a Malvern Panalytical DLS and Zetasizer Ultra. The amine and thiol groups on the surface of AP-SIONPs (1 mg) or MP-SIONPs (1 mg) were quantified by using ninhydrin or Ellman’s reagent, respectively. Briefly, to quantify the amines present on the surface of the AP-SIONPs, 1 mL of ninhydrin (15 mg/mL, ethanol) was added to 4 mL of ethanol containing 1 mg of AP-SIONPs. After reacting for 15 min at 80 °C, the solution was centrifuged at 13,000 rpm for 15 min. The supernatant was then injected into a quartz cuvette and analyzed with a Nanodrop UV–vis spectrophotometer, and the absorbance at 575 nm was used to find the concentration. A calibration curve was generated to find the unknown concentration of the sample by using a similar technique with different amounts of APTES (0–100 μg APTES in 4 mL of ethanol). For quantifying thiols, 50 μL of Ellman’s reagent (4 mg/mL, phosphate buffer, pH = 8.8) was added to 2.95 mL of phosphate buffer and DTT-treated MP-SIONPs. After reacting for 15 min at room temperature, the absorbance at 412 nm was used to calculate the concentration of thiols. A calibration curve was generated to find the unknown concentration of the sample by using a similar technique with different amounts of MPTES (1–75 μg MPTES in 3 mL of phosphate buffer).

#### 2.3.3 Metal Affinity Analysis

The Au- or Ag-decorated T-SIONPs, AP-SIONPs, and MP-SIONPs (150 μg) were digested in concentrated HCl or HNO_3_ (15 mL) in a 50 mL volumetric flask and gently boiled until solution was evaporated to about 5 mL (4–6 h). Considering Au is more soluble in HCl, HCl was applied to the samples for digestion and further suspension for Au quantification. For Fe and Ag quantification, HNO_3_ was applied for digestion and further suspension. The digested sample was resuspended in 50 mL 2% HCl or HNO_3_. Using a syringe, the solution was filtered through a sterile 0.22 μm Millipore Express PES Membrane Filter Unit. Then 250 μL of the sample was injected into a secondary vial to make a total of 5 mL 2% acidic solution. Au, Ag, and Fe, standards were prepared in the corresponding acidic solutions to generate a calibration curve and used as a reference when determining the quantity of the elements for each sample. The samples and standards were analyzed under ICP-OES (Optima 3000, Agilent). Samples were introduced into plasma by a peristaltic pump and discharged as an aerosol suspended in an argon gas. The data acquisition was performed in triplicates with the torch assembly in the axial mode. The default acquisition parameters used are RF = 1.2 kW; auxiliary gas flow = 1 L min^–1^, nebulizer gas flow = 0.7 L min^–1^; plasma flow = 12 L min^–1^, pump speed = 12 rpm; stabilization time = 15 s; sample uptake time = 25 s; rinse time = 30 s and analytical lines: 238.204 nm (Fe), 328.068 nm (Ag), and 267.594 nm (Au).

#### 2.3.4 Dark Field Microscopy

NPs were diluted ten times and 20 μL of the sample was dropped on a glass slide and covered with a glass cover fixed in place by parafilm. The samples were viewed with an Xplora Plus Raman Microscope/Olympus BX41 (60X lens). The samples were illuminated with a halogen lamp (Fuber-Lite DC 950) at full power. Images of the scattering samples were taken with a Horiba Cytoviva camera and a single particle analysis was performed with program ENVI 4.8. using three regions of interest (shown as red dots in Figure 4) and averaging the spectral profile.

#### 2.3.5 Atomic Composition Analysis Using EDS-SEM

Samples (∼0.1 mg/mL, 7 μL) were dropcasted on a silica wafer and dried at room temperature for 24 h. Three replicates of each sample were then imaged, and elemental mapping was performed with a Phenom XL G3 Desktop SEM instrument at 15 k× magnification and 15 kV high voltage. To determine homogeneity in decoration, the atomic concentration (%) as provided by the instrument for Fe, Au, and Ag was averaged out of 12 images per replicate, and graphed for comparison.

#### 2.3.6 Treatment of Bacteria

Using a 96-well round-bottom microwell plate, *E. coli* (K12 MG1655) or *S. aureus* (Xen 40) fixed at 5 × 10^5^ CFU/mL under Mueller Hinton Broth (MHB) was added to each well. Then, AgMP-SIONPs, AP-SIONPs, AuAP-SIONPs, and MP-SIONPs were suspended in autoclaved water were added to independent wells to provide final concentrations of 1,000, 500, 250, 125, 62.5, 31.25, and 15.63 μg/mL providing a water to MHB ratio <10%. In a separate well, water was used as a negative control at the same time as the NP treatment in a different well. Each plate contained at least three technical replicates and two biological replicates for each sample treatment. This experiment was repeated at least two times with three different batches of samples, which accumulated to four biological replicates and 12 averaged technical replicates. The plate was covered with a sterile sealing film before replacing the well plate lid. The plate was then placed in the shaker incubator set at 37 °C and 200 rpm for 18 h. Afterward, the plate was removed from the incubator shaker and placed on top of a magnet to separate the SIONPs for 5–10 min. The supernatant was then extracted and injected into a separate flat-bottom 96-well plate and the optical density at 600 nm was read with a Tecan Spark Multimode Microplate Reader. The results were background subtracted with the measurement of sterile MHB. The bacterial inhibition percentage for each sample and dosage was measured with eq 1. To determine MBC, the aliquots of the supernatant after treatment were dropped on LB agar plates and incubated overnight.

1 $$\left[\frac{\left({OD}_{control}-{OD}_{sample}\right)}{{OD}_{control}}\right]\times 100$$

#### 2.3.7 Capture Efficiency of IONPs

Following treatment with *E. coli* or *S. aureus* similar to Section 2.3.5., the SIONPs were magnetically separated and a small aliquot of the supernatant treated with AgMP-SIONPs, AP-SIONPs, or MP-SIONPs at 125, 62.5, or 31.25 μg/mL was serially diluted in a broth and dropped on a 100 mm plate containing agar with either Luria broth (LB) or brain heart infusion (BHI) broth for bacterial enumeration. Then the supernatant was completely removed, and the pellet was washed three times with autoclaved sterile water and suspended in autoclaved water to return to its initial concentration. A small aliquot of the pellet was serially diluted in a culturing broth and dropped on a 100 mm plate like the process for the supernatant. Negative control was treated in a similar manner to account for any nonmagnetic retrieved bacteria and applied to eq 2 to determine the capture efficiency of the three types of IONPs.

2 $${\left[\frac{\mathrm{Log}{\left(\frac{CFU}{mL}\right)}_{pellet}}{\mathrm{Log}{\left(\frac{CFU}{mL}\right)}_{pellet}+\mathrm{Log}{\left(\frac{CFU}{mL}\right)}_{supernatant}}\right]}_{sample}-{\left[\frac{\mathrm{Log}{\left(\frac{CFU}{mL}\right)}_{pellet}}{\mathrm{Log}{\left(\frac{CFU}{mL}\right)}_{pellet}+\mathrm{Log}{\left(\frac{CFU}{mL}\right)}_{supernatant}}\right]}_{control}\times 100$$

#### 2.3.8 Statistical Analysis

Graphs and statistical analyses were performed using OriginPro 2024 (Academic Version). Statistical significance between the interaction of elements (Au and Ag) and ligand was assessed by two-way analysis of variance (ANOVA). All the statistical analyses were performed with at least three replicates, α = 0.05, and reported as stars assigned to the following p values: *p < 0.05, **p ≤ 0.01, and ***p ≤ 0.001.

## 3 Results and Discussion

### 3.1 Synthesis and Structural Characterization of SIONPs

IONPs were synthesized based on a modified coprecipitation method provided by Besenhard et al.^31^ and Osial et al.^32^ Within their crystalline structure, IONPs are prone to oxidation in acidic conditions, temperature changes, and displacement from other divalent ions (e.g., Mg^2+^) all of which change the lattice structure and could lead to decreased or even complete loss of magnetization.^33^ To solve this issue, silica coating of the IONPs can be incorporated in the synthesis.^34,35^ Therefore, in this work, encapsulation of IONPs was achieved by coating with APTES, MPTES, and TEOS, providing the three types of SIONPs. TEOS was added as an intermediate step for APTES and MPTES to optimize silica encapsulation and controllable linkage for further functionality.^36,37^ As stated in the introduction, this one-pot method has never been successfully achieved for further decorating with an antibacterial metal. To our knowledge, the synthesis of SIONPs has only been reported with the use of organic solvents, high temperature, high pressure, or with intermediate washing steps.^24–28^

This synthesis utilizes the basic conditions needed for the hydrolysis and precipitation of magnetite by adding the silica precursor directly into the aqueous solution after magnetite has been formed. In doing so, this ensures that the bare IONPs are not exposed to external factors such as additional washing steps, which could introduce oxidizing conditions and degrade the IONPs, resulting in reduced magnetization. After the synthesis is complete, the washing steps with water and ethanol via magnetic separation ensure complete removal of unwanted biproducts such as IONPs with poor magnetic properties, silica biproducts, and unreacted reagents.

Figure 2A shows the PXRD patterns of IONPs with and without silica coating. The characteristic peaks of magnetite are listed in Figure S1. These patterns agree with other literature reports^38–40^ and an online database (RRUFF ID: R061111). After silica coating, the PXRD results confirm that magnetite is still present in the material. Additionally, EDS was performed with the captured TEM images (Figures S2A–C), which shows a substantial increase in iron intensity compared to copper, as well as increased silica intensity. Therefore, these results confirm an overall higher iron content after silica encapsulation. The magnetic properties of the IONPs and SIONPs were tested in solution as shown in Figure S3. The images of the IONPs and SIONPs suspended in water (0.1 mg/mL) in the presence of a magnet demonstrate the magnetic features of the NPs.

**Figure 2:**
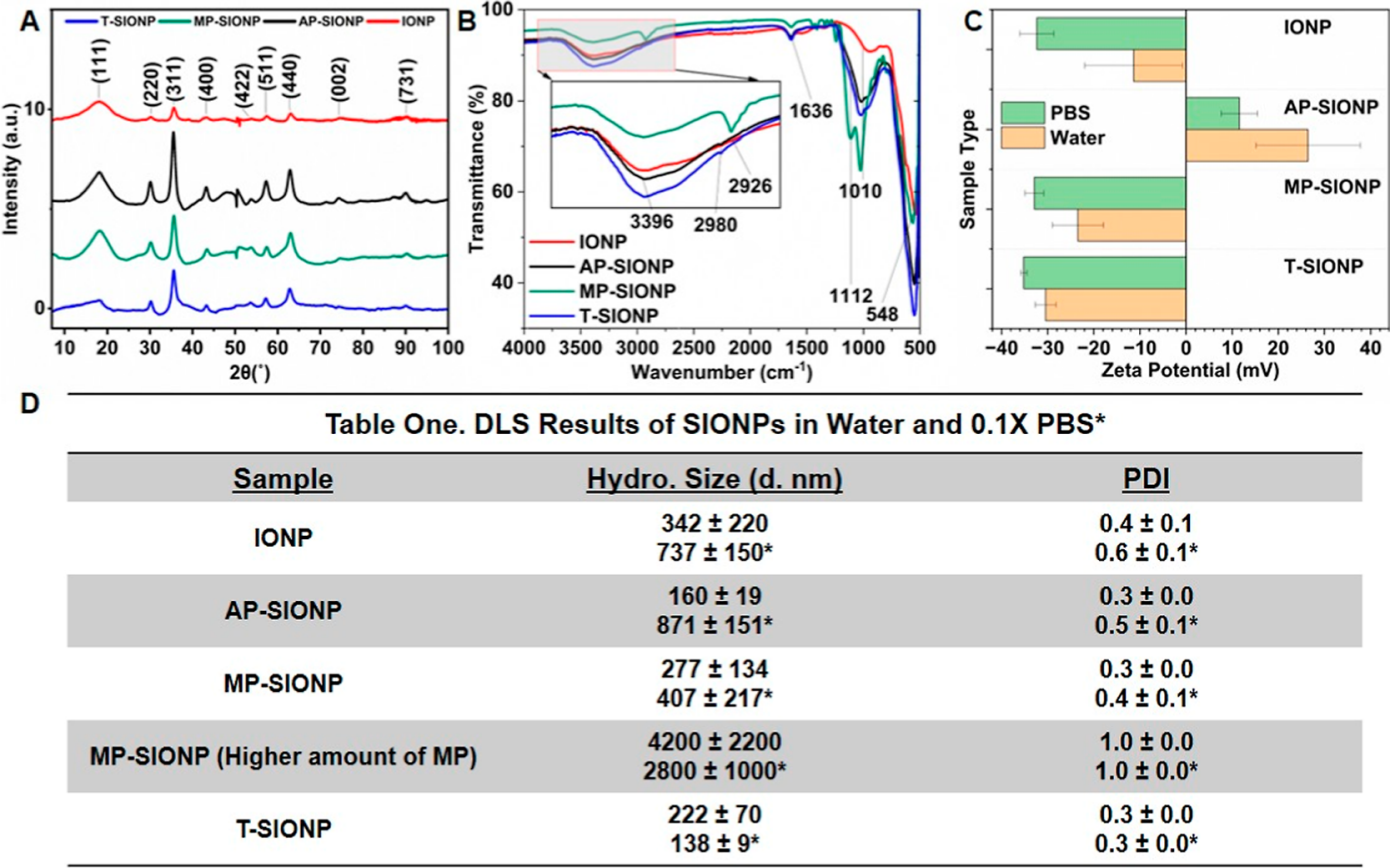
PXRD patterns of IONPs (red), AP-SIONPs (black), T-SIONPs (blue), and MP-SIONPs (green) (A). FTIR spectra of IONPs (red) and SIONPs (green, blue, and black) (B). The zeta potential of the IONPs and SIONPs in water (orange) and 0.1× PBS (green) are shown (C). The hydrodynamic diameter and polydispersity index (PDI) that are provided by the instrument are listed in Table One with numerical values associating to water and 0.1× DPBS* (D).

### 3.2 Silica Coating and Ligand Functionalization

The FT-IR spectra of the four different samples (Figure 2B) confirms the presence of the following vibrational bands: Fe–O (548 cm^–1^),^41^ O–H (3396 and 1636 cm^–1^),^42^ Si–O–Si (1112 cm^–1^), Si–O (1010 cm^–1^), and C–H (2926–2858 cm^–1^).^43^ These vibrational bands confirm that not only is Fe present, but Si has encapsulated the SIONPs with the applied sol–gel process. However, the characteristic vibrational bands for thiols (2500 cm^–1^) and amines (1575 cm^–1^) for MP-SIONPs and AP-SIONPs, respectively, were not observed most likely due to the low concentration of these groups in the SIONPs (Figure S4). To further investigate the question of whether free amine or thiol ligands were present on the surface of the SIONPs, colorimetric assays were performed using ninhydrin and Ellman’s reagent, respectively.

Ninhydrin is a very strong electrophile ready to react with nucleophiles such as amines to form a purple-colored molecule, Ruhemann’s purple.^44^ The molecule absorbs light at 575 nm and can easily be detected with UV–vis spectrophotometry.^45^ Ellman’s reagent (5,5′-dithiobis­(2-nitrobenzoic acid)) (DTNB) is commonly used for the quantification of thiols in cellular processes.^46^ DTNB is reduced at the disulfide bond under the presence of thiolates (the analyte) and forms 5-thio-2-nitrobenzoic acid (TNB), which has a high extinction coefficient (14,150 M^–1^ cm^–1^) under aqueous conditions at pH 7–8.^47^

The result of the calibration curves for APTES and MPTES are shown in Figures S5A and S5C. The AP-SIONPs show a concentration of 143.9 ± 55.6 μg APTES/mg AP-SIONPs (*n* = 3) (Figure S5B). The large standard deviation of the APTES coating signifies variability in the number of amines chemically available present on the surface of the SIONPs throughout batches; however, the colorimetric assay confirms the successful coating of amine-functionalized silica. Figure S5D shows an optimal MPTES concentration at 21.6 ± 1.5 μg/mg MP-SIONPs (*n* = 3) after an ideal 6 h of reaction with DTT (Figure S6A). Efforts were made to increase the number of thiols on the MP-SIONPs by performing longer reduction times with DTT and even separately increasing the amount of MPTES added during IONP synthesis. Unfortunately, the increased MPTES in synthesis led to less colloidally stable MP-SIONPS (Figure 2D) while the increased DTT reactions reduced the amount of thiols (Figure S6A). Additionally, T-SIONPs were used for each colorimetric assay to confirm the accuracy. It is shown that amines and thiols were found at minimal concentrations for T-SIONPs, which is explained in more detail in the ESI (Figure S6B).

In Figure 2D, DLS values for all three SIONPs show that the silica contributes to enhanced colloidal stability which supports literature reports.^48^ Due to the future potential of applying these SIONPs to either water or clinical treatment, the DLS was compared in water and PBS (Figures S7A,B). In water, the IONPs have a higher polydispersity index (PDI) than the SIONPs. AP-SIONPs and T-SIONPs have the least PDI (<0.3) while MP-SIONPs show a right-skewed distribution but still a lower hydrodynamic diameter compared to IONPs. The colloidal stability of these materials in water follows this trend: AP-SIONPs > T-SIONPs > MP-SIONPs > IONPs. In the case of PBS, the results show that the AP-SIONPs tend to aggregate and have a bimodal PDI while T-SIONPs are the most colloidally stable (Figure S7B). IONPs in 0.1X PBS appear to have the second highest hydrodynamic diameter, but the PDI is the highest (0.6 ± 0.1). MP-SIONPs in PBS appear to show a small degree of bimodal distribution; moreover, the hydrodynamic diameter and PDI are lower than both the IONPs and AP-SIONPs. The colloidal stability of these materials in PBS follows this trend: T-SIONPs ≫ MP-SIONPs > AP-SIONPs > IONPs.

Figure 2C shows the zeta potential values of these NPs in the same solvents. The contribution of thiols and hydroxides increases the overall negative surface charge, and the amines increase the positive charge with respect to IONPs. This increases the stability of the IONPs through electronegative repulsions.^49^ By first studying the IONPs and SIONPs in water (pH ∼ 5), it is evident that the IONPs (−11 ± 10 mV) are greatly less charged than the AP-SIONPs (26 ± 11 mV), T-SIONPs (−30 ± 2 mV), and MP-SIONPs (−23 ± 5 mV). However, when the samples are suspended in a solvent with a higher ionic strength like PBS, the zeta potential drastically decreases for all samples. Zeta potential differing in varying solvent ionic strengths has been commonly reported in the current literature and can be explained by the ion’s ability to associate with the NP at the slipping plane and shield the surface charge thus reducing the zeta potential.^50^ Unfortunately, this causes the AP-SIONPs to lose their electrostatic repulsive force (11 ± 4 mV) and therefore lose their colloidal stability. However, the colloidal stability related to NPs involves more than electric repulsion since the zeta potential for MP-SIONPs (−33 ± 2 mV), T-SIONPs (−35 ± 1 mV), and IONPs (−32 ± 4 mV) greatly decreased but still show decreased colloidal stability when the DLS data is compared. Herein, two main effects are playing a role, the T-SIONPs have a hydration layer which provides strong hydration repulsive forces;^51^ however, the MP-SIONPs provide hydrophobicity, which is seen in the decreased water and increased hydrocarbon vibrational bands seen in the FTIR spectrum.^52^ Therefore, the hydrophobic effect provided by the MP-SIONPs explains the higher PDI in both solvents with respect to the other two SIONPs. Silica coating nevertheless provides a higher degree of colloidal stability compared to noncoated IONPs.

### 3.3 Synthesis of Silver (Ag)- or Gold (Au)-Decorated SIONPs

Once the three SIONPs were synthesized and characterized, the NPs were then decorated with antibacterial metals (Au or Ag) under aqueous conditions and with the use of glucose as a reducing agent. The amount of metal present within a given amount of NP sample was determined with inductively coupled plasma-optical emission spectroscopy (ICP-OES). The homogeneity of the metallic decoration of SIONPs was visualized using dark field microscopy (DFM). The mean elemental concentration of Au and Ag found in the six samples were quantified by ICP-OES (Figure 3A). The concentration curves and graph to show the overall elemental content (Au, Ag, and Fe) are shown in Figures S8A–D. In general, Au and Ag have an affinity for all three ligands. However, the amount of Ag was calculated to be significantly higher for MP-SIONPs (160 ± 23 μg/L) than AP-SIONPs (72 ± 36 μg/L) and T-SIONPs (80 ± 18 μg/L). On the other hand, Au was found to be at a higher concentration for AP-SIONPs (79 ± 18 μg/L) and T-SIONPs (64 ± 14 μg/L) compared to MP-SIONPs (24 ± 0 μg/L).

**Figure 3:**
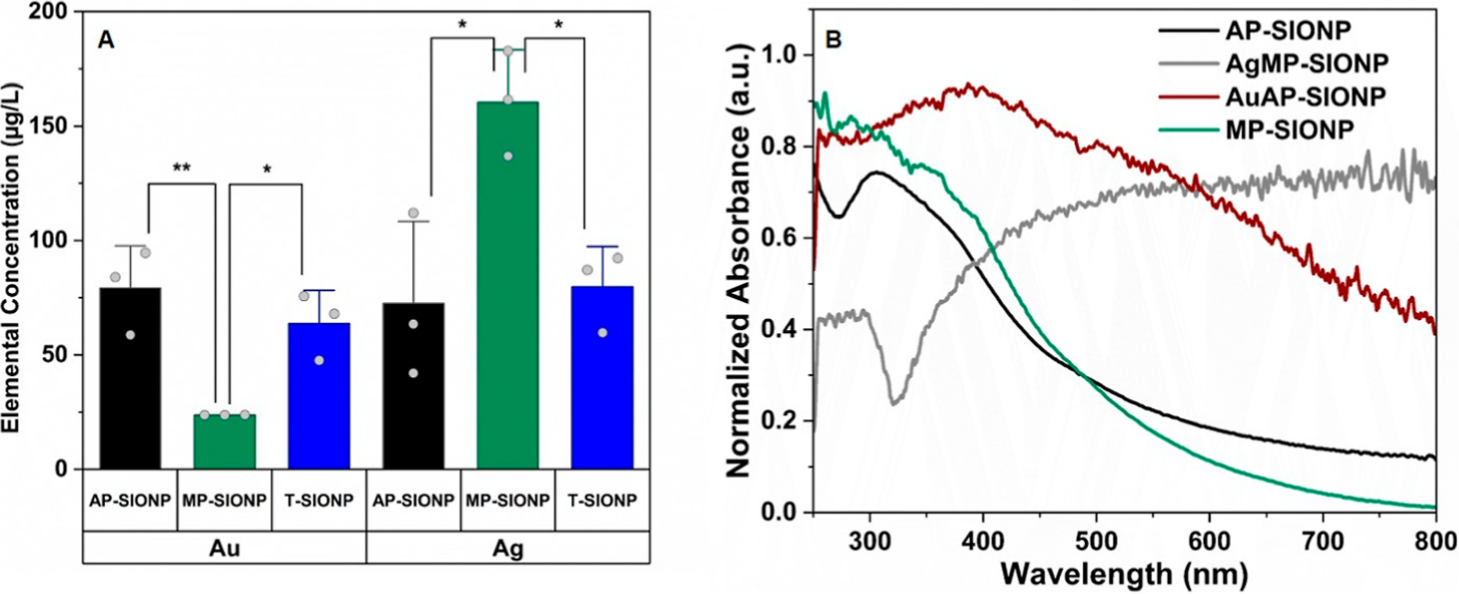
Elemental concentration (μg/L) of Au (left) and Ag (right) found coated on AP-SIONPs (black), MP-SIONPs (green), and T-SIONPs (blue). The interaction between the element and sample was found to be statistically significant (*p* = 0.00151). Scattering plots of the mean populations that were generated by Two-Way ANOVA are provided in Figure S9.

To account for these results, it is important to understand the chemical affinity of the metallic precursors, HAuCl_4_ (Au^3+^) and AgNO_3_ (Ag^+^), which behave as Lewis acids and favor bonding with ligands that behave as Lewis bases. However, the chemical properties of these two metals are different due to their charge (Ag^+^ and Au^3+^), and the interaction of this metal–ligand can vary. The theory that can predict the metal–ligand interaction is known as the hard/soft acid/base (HSAB) theory.^11,29,30^ In general, it is theorized that there are two classes of metals and ligands (hard and soft) which is governed by the magnitude of the charge, ionic size, and polarizability. It is more favorable for these metals and ligands to bind when they fall under the same class. In our study, HAuCl_4_ affords Au^3+^, which is considered a hard acid due to its high charge/small ionic radius. On the contrary, the precursor AgNO_3_ affords Ag^+^ which has a higher ionic radius and lower charge with respect to the Au^3+^ and, therefore, would be considered a soft acid. Since thiols are considered soft bases, and amines and hydroxides are regarded as hard bases, our findings agree with the HSAB and confirm that it is applicable for selecting the optimal ligands for synthesis of metallic NPs. Lastly, one of each Ag-and Au-decorated SIONPs were analyzed under a spectrophotometer to show the complete reduction of the antibacterial metal on the surface (Figure 3B). Both Ag- and Au-decorated SIONPs provided a very broad peak that increased at ∼400 nm compared to the SIONPs. Typically, absorption for Au and Ag NPs are expected to absorb at wavelengths 500 and 400 nm, respectively, depending on the shape and size.^53,54^ However, it is possible that the already aggregated SIONPs in solution during decoration contributed to the broadening of the absorption.

Noble metals such as Au and Ag are advantageous for qualitative analysis due to their plasmonic components at the nanoscale size. Once the plasmonic NP is bombarded with light, the conduction electrons constructively oscillate and, in turn, provide intense scattering of light.^55^ This scattering can be observed with DFM and allows for the flexibility to identify and locate plasmonic NPs. Moreover, DFM provides single-particle scattering profiles confirming the decoration of NPs by visualizing the change in the scattering intensity and wavelength.^56,57^ Additionally, the color at which the NPs scatter can be visualized in the images and provide proof of composition heterogeneity and homogeneity. To optimize the antibacterial treatment, the IONPs decorated with the highest content of metal (AuAP-SIONP and AgMP-SIONP) and the lowest content of metal (AuMP-SIONP and AgAP-SIONP) were analyzed and compared using TEM and DFM for further qualitative analysis (Figures 4 and S10). Using AgNPs as a reference (Figure 4C), AgMP-SIONPs (Figure 4B) show more uniform scattering compared with AgAP-SIONPs (Figure 4A). As shown, the majority of AgAP-SIONPs scatter white light, with a select few scattering yellow light, the latter observation being similar to the behavior of AgNPs. AgMP-SIONPs scatter a yellow light throughout the sample, signifying that the MP-SIONPs are homogeneously decorated with Ag. The TEM image of the AgAP-SIONPs (Figure 4D) captures the heterogeneity of the sample because of the appearance of large, cubic NPs surrounded by smaller spherical NPs. However, the AgMP-SIONP TEM image (Figure 4E) shows a much more monodisperse sample due to the appearance of small, spherical NPs.

**Figure 4:**
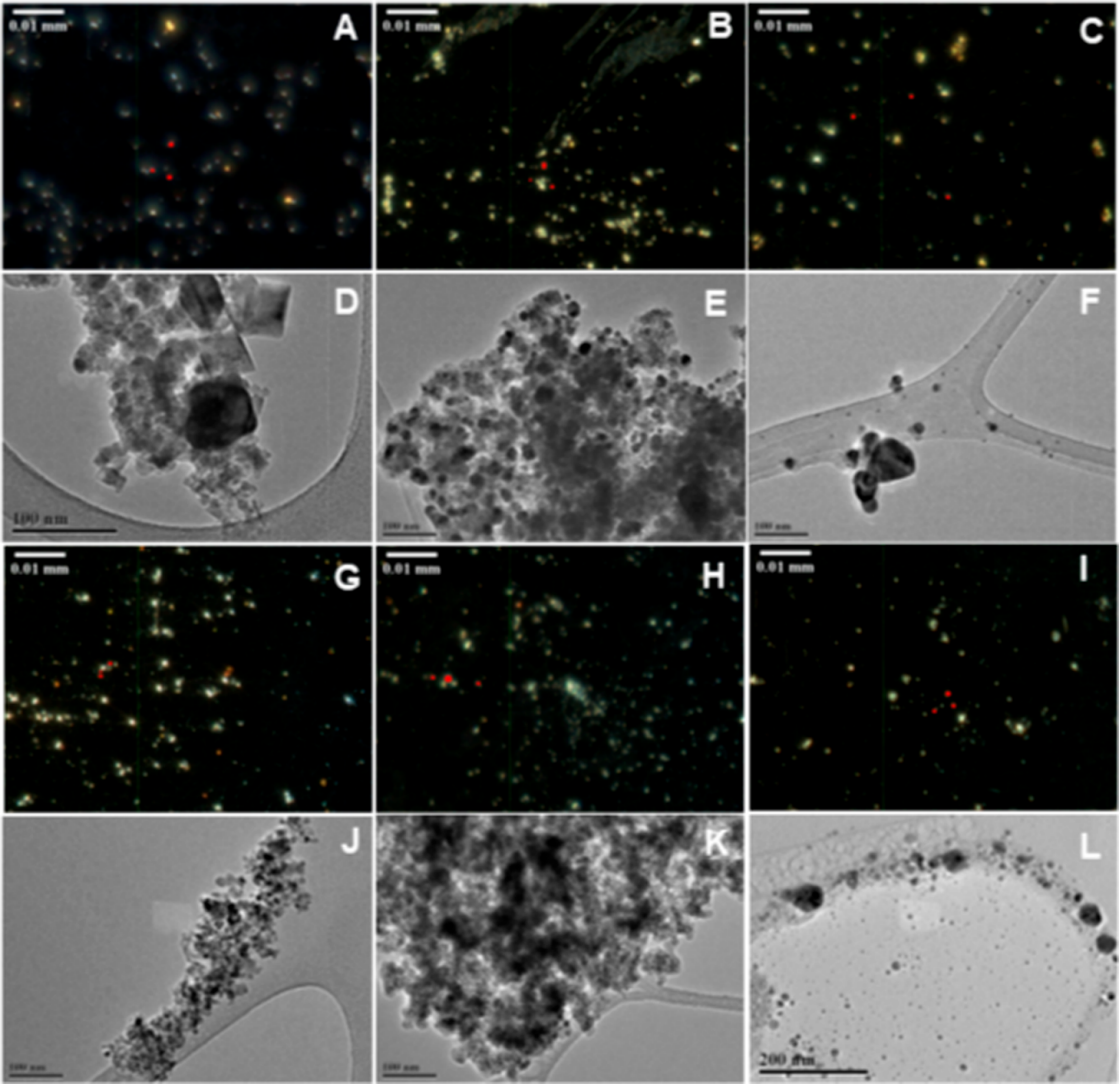
DFM (scale: 0.01 mm) (A–C) and TEM images (D–F) of AgAP-SIONPs (A,D), AgMP-SIONPs (B,E), and AgNPs (C,F). DFM (G–I) and TEM images (J–L) of AuAP-SIONPs (G, J), AuMP-SIONPs (H, ), and AuNPs (I,L). All TEM images were taken at 80k× magnification (scale:100 nm) except AuNPs (L) magnification (scale: 200 nm).

On the contrary, the AuSIONPs show a more homogeneous decoration throughout the sample no matter the ligand (Figure 4G–L). Since Au^3+^ is considered a hard acid, it was expected that there would be heterogeneity in the scattering of AuMP-SIONPs. However, the DFM and TEM images for AuMP-SIONPs showed a homogeneous scattering of Au (Figure 4H) and morphology (Figure 4K). As an ion, Au has a range of oxidation states which can change its classification as a hard or soft acid.^58^ Therefore, we hypothesize that the homogeneous decoration for all ligands is due to the changes in the oxidation states of Au, changing its level of hardness/softness throughout the synthesis. The scattering of the AuSIONPs can be compared by using AuNPs as a reference (Figure 4I). Lastly, as expected, the scattering and morphology of Au for AuAP-SIONPs (Figure 4G,J) were homogeneous throughout the material. The scattering profiles for all four samples are much higher than the control (T-SIONP) (Figure S10), which indicates successful decoration with a plasmonic element such as Au and Ag. It is important to point out that the intensity is also higher than those of the AuNPs and AgNPs alone, most likely due to the aggregation of the SIONPs. Overall, based on these results together with ICP-OES data, we selected AgMP-SIONPs and AuAP-SIONPs as the optimal NPs to use in the antibacterial experiments.

To confirm the accuracy of using DFM to determine the homogeneity of SIONP decoration with either Au or Ag, elemental mapping was performed, and the atomic concentration (%) was averaged and compared with each nanomaterial as shown in Figure S11. As observed in the DFM images for Ag (Figure 4A), regions with varying amounts of Ag are present, resulting in a large standard deviation for AgAPSIONPs (17 ± 13%). In contrast, AgMP-SIONPs exhibit more consistently higher amounts of Ag decoration throughout the images (20 ± 5%), leading to a reduced standard deviation. For Au decoration, elemental mapping confirmed a generally lower level of metallic decoration compared to Ag-decorated SIONPs. However, AuAP-SIONPs display a higher degree of Au decoration (7.1 ± 3.1%) than AuMP-SIONPs (2.8 ± 0.5%). These results indicate that the uniformity of metallic decoration depends on the choice of ligand.

### 3.4 Antibacterial Analysis

Lastly, the antibacterial properties of the metallic SIONPs were investigated by application to *E. coli* (K12 strain) or *S. aureus* (Xen 40) bacteria. Figure 5A shows the inhibition of *E. coli* following 18 h of exposure to MP-SIONPs, AP-SIONPs, AgMP-SIONPs, or AuAP-SIONPs. No statistically significant differences were observed among the four nanoparticle formulations at concentrations of 31.25 and 15.63 μg/mL. However, at 62.5 μg/mL, AgMP-SIONPs exhibited significant antibacterial activity (92.90 ± 5.03%), significantly higher than AP-SIONPs (5.8 ± 3.3%), AuAP-SIONPs (25.5 ± 2.1%), and MP-SIONPs (3.3 ± 3.8%), identifying this concentration as the MIC. In contrast, AuAP-SIONPs show dose-dependent inhibition at 250 (42.5 ± 21.6%), 500 (79.8 ± 9.2%), and 1000 μg/mL (82.3 ± 12.6%), but without reaching the MIC. Both AP-SIONPs and MP-SIONPs showed no significant bacterial inhibition for the Gram-negative bacteria. When tested against *S. aureus* (Figure 5C), the MIC for AgMP-SIONPs increased to 500 μg/mL (94.2 ± 2.3%), with notable reduction already observed at 250 μg/mL (68.4 ± 27.8%), indicating reduced antibacterial potency compared to *E. coli*. AuAP-SIONPs showed no significant inhibition against *S. aureus*. Interestingly, both MP-SIONPs and AP-SIONPs exhibited better antibacterial activity than AuAP-SIONPs at 1000 μg/mL (65.0 ± 12.3% and 45.6 ± 3.3%, respectively). Among all nanomaterials, only AgMP-SIONPs achieved a minimum bacterial concentration (MBC) of 62.5 μg/mL against *E. coli*, while no MBC was observed for *S. aureus* (Figure S12).

**Figure 5:**
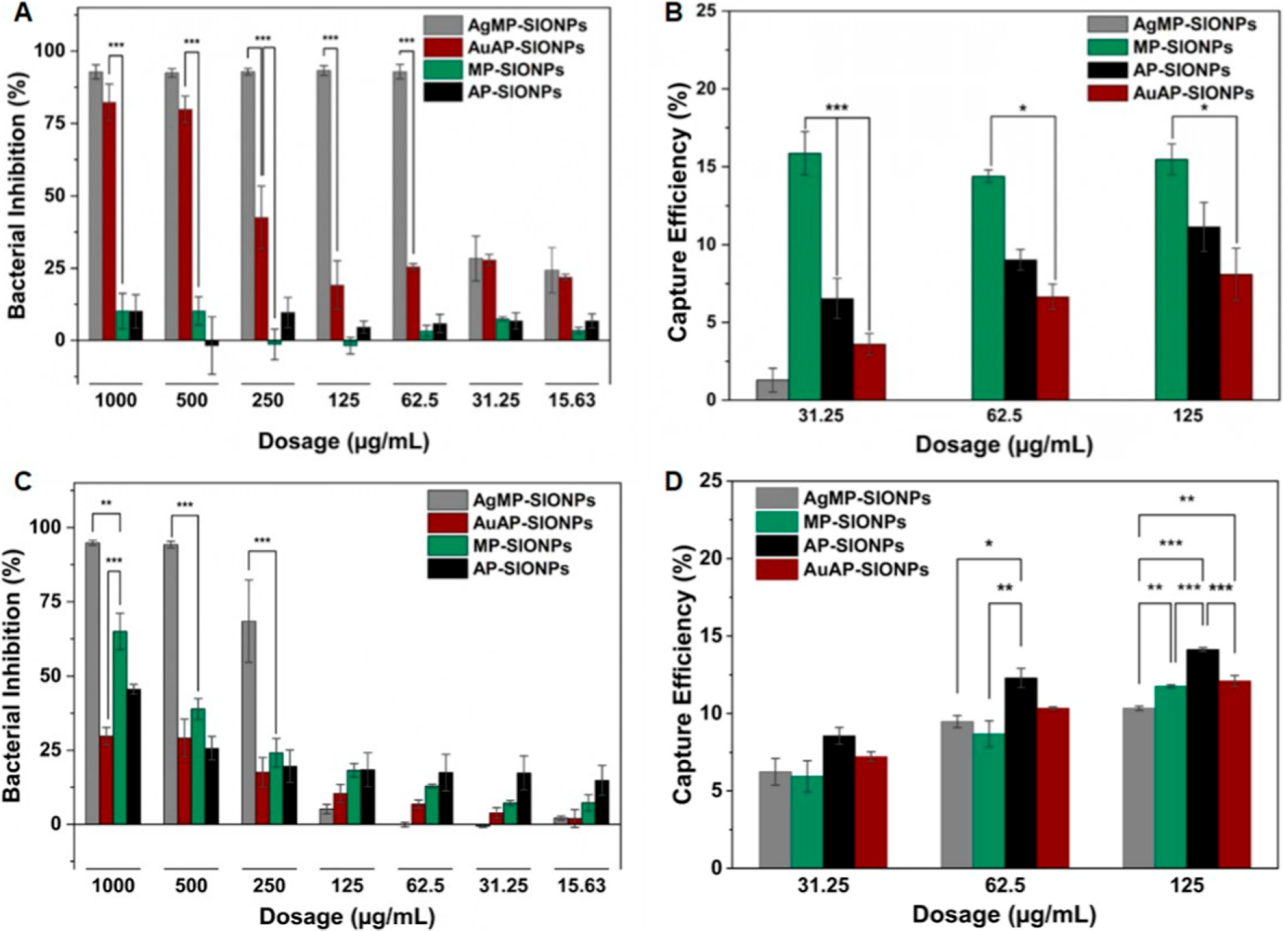
Bacterial reduction and capture efficiency of (A,B) *E. coli* K12 and (C,D) *S. aureus* Xen40 strain for the metallic coated SIONPs (AgMP-SIONP (silver) and AuAP-SIONP (red)) and the complementary SIONPs (MP-SIONP (green) and AP-SIONP (black)) are shown.

Previous studies have demonstrated that the release of Ag^+^ ions plays a critical role in the inhibition of bacteria, which accounts for the strong bactericidal activity of AgNPs.^11,13^ On the contrary, AuNPs exhibit antibacterial activity primarily through their affinity for hydroxyl and thiol groups present in bacterial membranes, including lipopolysaccharides, phospholipids, and thiol-containing proteins; however, this mechanism generally results in comparatively lower antibacterial efficacy.^7^ The results of the present study are consistent with these findings, as AgMP-SIONPs exhibited significantly higher antibacterial activity than AuAP-SIONPs against both *E. coli* and *S. aureus*. Interestingly, AgMP-SIONPs demonstrate greater effectiveness against *E. coli* than against *S. aureus*. This difference can be attributed to structural variations in the cell envelopes of Gram-negative and Gram-positive bacteria. Gram-negative bacteria possess an outer membrane composed of lipopolysaccharides, followed by a relatively thin peptidoglycan layer.^59^ In contrast, Gram-positive bacteria are characterized by a much thicker peptidoglycan layer within their cell walls.^60^ This structural disparity facilitates the penetration of Ag + ions into Gram-negative bacteria, thereby enhancing their susceptibility to AgMP-SIONPs.^61,62^

To further elucidate (i) SIONP-bacteria interactions and their contribution to antibacterial activity and (ii) the feasibility of magnetic retrieval, capture efficiency was evaluated for MP-SIONPs, AP-SIONPs, AgMP-SIONPs, or AuAP-SIONPs with both *E. coli* (Figure 5B) and *S. aureus* (Figure 5D). All nanoparticles demonstrated measurable capture efficiencies, except for AgMP-SIONPs in the case of *E. coli*, likely due to the increased susceptibility of this strain to Ag. Interestingly, MP-SIONPs and AP-SIONPs showed enhanced capture efficiencies toward *E. coli* and *S. aureus*, respectively. For *E. coli*, MP-SIONPs exhibited consistent capture efficiencies across concentrations: 31.2 (15.9 ± 2.4%), 62.5 (14.4 ± 0.7%), and 125.0 (15.5 ± 1.7%) μg/mL. In contrast, AP-SIONPs demonstrated concentration-dependent capture efficiencies against both *E. coli* and *S. aureus*. This trend is most likely due to their positive charge which favors the electrostatic interaction of the NP and the bacteria outer membrane. Both Gram-negative and Gram-positive bacteria contain a negatively charged outer membrane due to the presence of negatively charged molecules (e.g., lipopolysaccharides and teichoic acids).^63,64^

## 4 Conclusion

In this work, we report a robust and tunable one-pot synthesis route for the preparation of silica-coated IONPs. This approach enables precise control over surface functionalization, as demonstrated through the successful incorporation of amine and thiols groups. These surface functionalities significantly influence the colloidal stability of the SIONPs, as confirmed by DLS results. Subsequent modification with Ag and Au was achieved and demonstrated by ICP-OES, DFM, TEM, and UV–vis spectroscopy. The observed metal–ligand interactions for the decoration of the nanoparticles were rationalized using the HSAB theory. Specifically, Ag^+^, a soft acid, exhibited preferential binding to thiol-functionalized MP-SIONPs (soft base), resulting in enhanced surface decoration. On the contrary, Au^3+^, characterized as a hard acid, showed improved interactions with amine- and hydroxyl-functionalized SIONPs (hard base). Antibacterial evaluation against *E. coli* and *S. aureus* revealed that AgMP-SIONP exhibit the highest efficacy, with a MIC/MBC of 62.5 μg/mL for *E. coli* and MIC of 500 μg/mL for *S. aureus*. Moreover, all four SIONP formulations demonstrated appreciable bacterial capture efficiency, highlighting their potential for combined antibacterial and separation applications. Overall, this study provides fundamental insights into ligand–metal interactions at the nanoscale and establishes a versatile platform for designing multifunctional antibacterial nanomaterials.

## Supplementary Material



## Notes

Powder diffraction of magnetite; EDS spectrum and TEM image for IONPs, AP-SIONPs, and MP-SIONPs; images of IONPs and SIONPs suspended in water under a magnetic field; FTIR spectra of silica precursors; calibration curve and UV–vis spectra for APTES quantification; calibration curve and UV–vis spectra for MPTES quantification; scattering plot of the amount of MPTES found in MP-SIONPs; DLS results of IONPs and AP-SIONPs, MP-SIONPs, and T-SIONPs in water and 0.1X PBS; calibration curves for silver, iron, and gold using ICP-OES; scattering plots of the mean populations of the samples (AP-SIONP, MP-SIONP, and T-SIONP) and elements (Ag and Au); scattering profile of AgNPs, AgAP-SIONPs, AgMP-SIONPs, and T-SIONP; scattering profile of AuNPs, AuAP-SIONPs, AuMP-SIONPs, and T-SIONP; atomic composition analysis for Ag, Au, and Fe using EDS-SEM; representative EDS-SEM images of the Ag- and Au-decorated nanomaterials; and images of agar plates after the supernatant post treatment (PDF)
