# Supplementary material for: Influence of Ligand Functionalization on the Synthesis of Metallic-Decorated Magnetic Nanoparticles for Antibacterial Treatment
Source: ACS Appl Nano Mater. 2026 Jun 24;9(26):12602–12. doi: 10.1021/acsanm.6c02039 (PMC13339128; doi:10.1021/acsanm.6c02039)
Supplement: Supplementary file 1 [file an6c02039_si_001.pdf]

Supporting Information

Influence of Ligand Functionalization on the Synthesis of Metallic-Decorated Magnetic Nanoparticles for Antibacterial Treatment

AUTHOR NAMES. Allison L. Stadick, Laura Scala, Juan L. Vivero-Escoto*

Email corresponding author: jviveroe@charlotte.edu

AUTHOR ADDRESS. University of North Carolina at Charlotte, 9201 University City Blvd, Charlotte, NC 28223

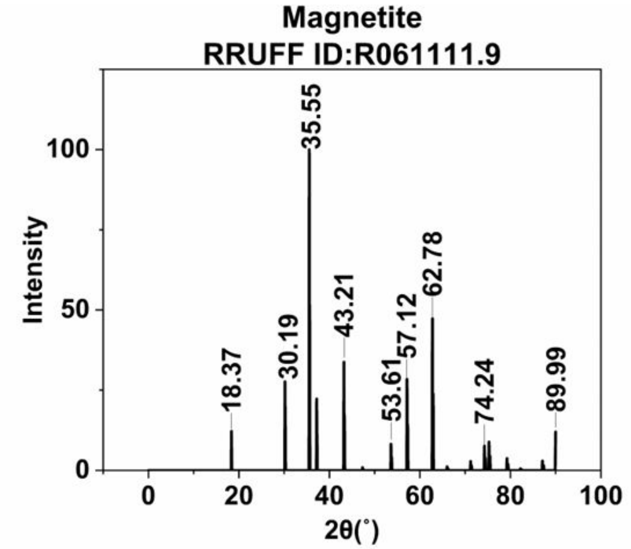

Table S1. PXRD Patterns

2θ(˚)	Phase
18.03	111
30.15	220
35.53	311
43.16	400
53.72	422
57.21	511
62.89	440
74.33	002
90.07	731

Figure S1. RRUFF powder diffraction characterization of magnetite (R061111).

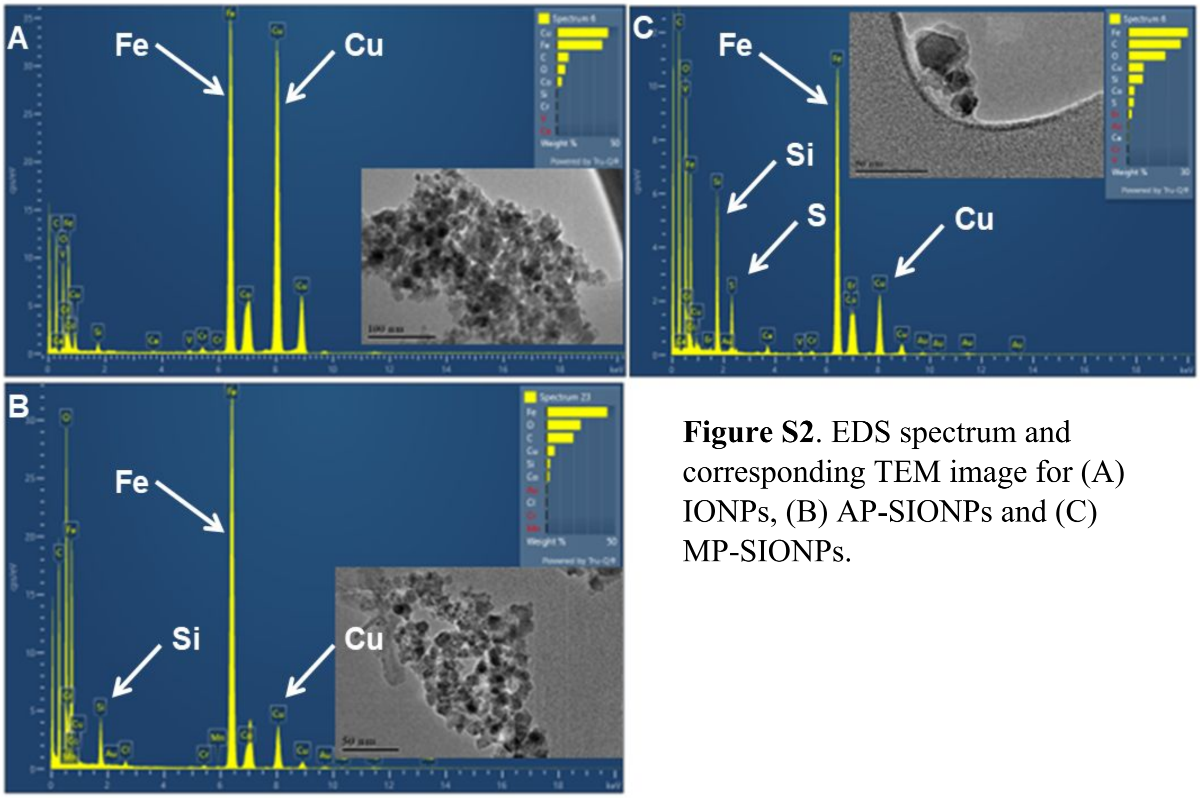

Figure S2. EDS spectrum and corresponding TEM image for (A) IONPs, (B) AP-SIONPs and (C) MP-SIONPs.

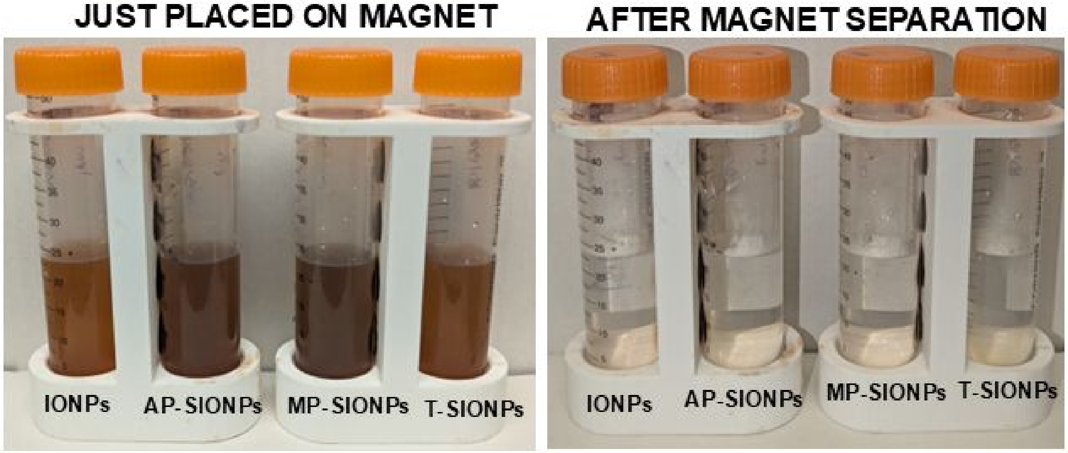

Figure S3. Images of IONPs and SIONPs suspended in water (0.1 mg/mL) and applied to a magnetic field.

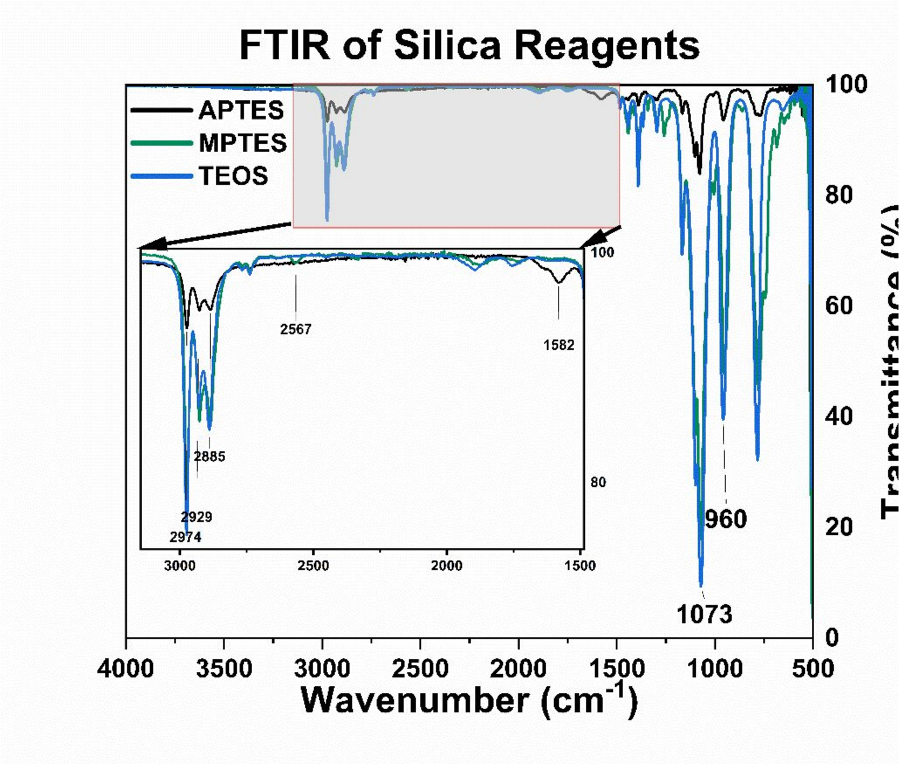

Figure S4. FTIR spectra of silica precursors APTES (black), MPTES (green), and TEOS (blue).

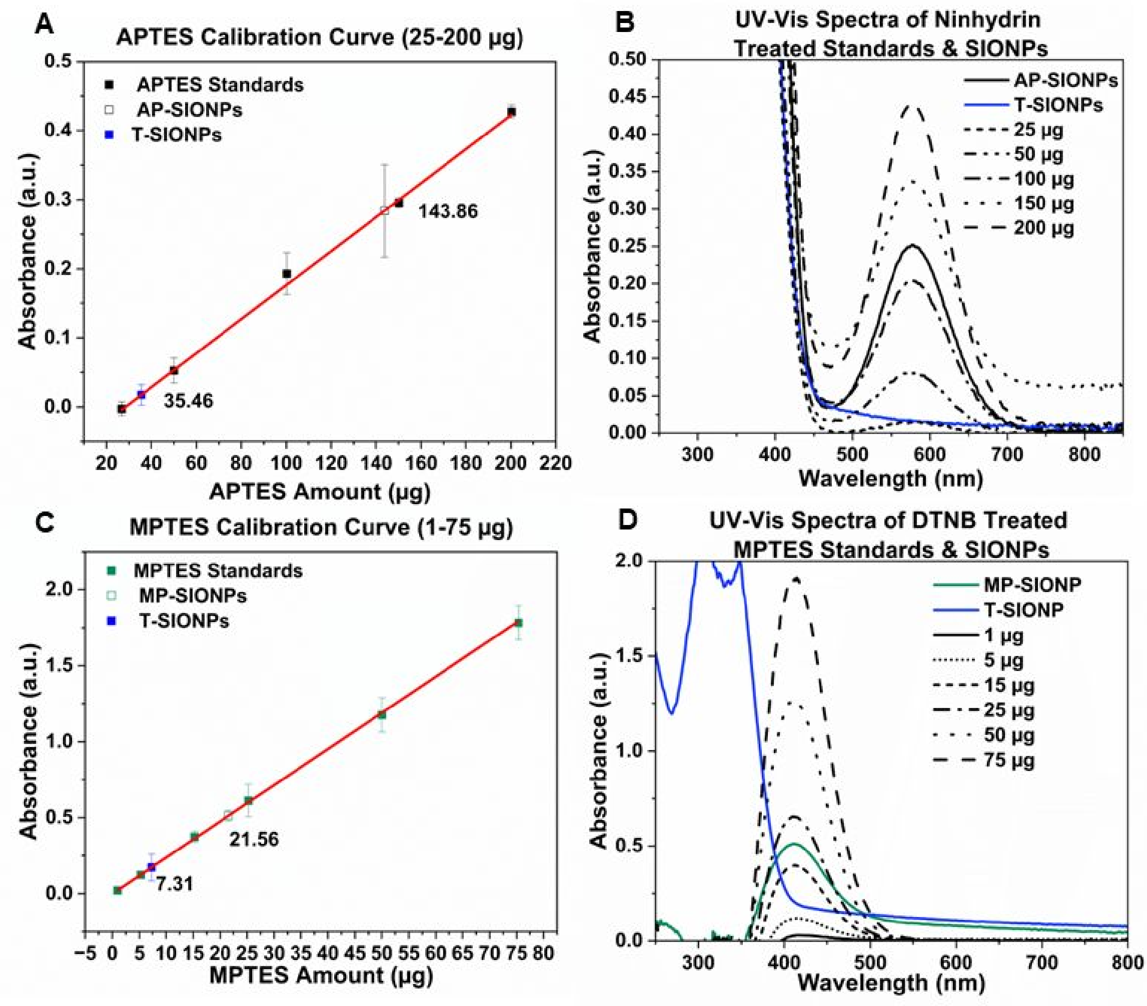

Figure S5. Calibration curve (A) and the corresponding UV-Vis Spectra (B) for APTES quantification with the use of ninhydrin. Calibration curve (C) and corresponding UV-Vis spectra (D) for MPTES quantification with the use of Ellman's reagent (C-D). T-SIONPs (blue square) was used as a control in both colorimetric assays.

Optimization of colorimetric assays: The T-SIONPs show a baseline with an absorbance between the lower two standards for both MPTES and APTES. Although this baseline is broad and does not actually distinguish a peak corresponding to the wavelength of interest, it still provides absorbance that must be considered in quantifying the ligands. Additionally, the stability of the silica layer and, therefore, leeching of the iron ions in solution are analyzed by performing similar experimental conditions with respect to the colorimetric assays but in the absence of the ninhydrin and Ellman's reagent (Figures **S6A-B**). The T-SIONPs show the highest to lowest degradation based on the absorbance at the two wavelengths of interest: water>>PBS>ethanol (80 ˚ C)>ethanol (25 ˚ C). There are literature reports that depending on the solvent, pH, and temperature, silica can undergo degradation.[59,60] Typically, the degradation of silica can occur under aqueous conditions due to the hydration of the silica surface which

consequently undergoes hydrolysis and ion-exchange and, in turn, leeching of silicic acid.[61] This would explain the high absorbance under nanopure water and PBS. However, it was interesting to see the absorbance was lower with PBS than water. It is unclear why this is, yet similar findings were reported with silica-based material.[62] Ethanol has a much lower pH (pH ~6) and, therefore, it favors the chemical stability of silica. However, when the temperature was raised to 80 ˚ C, the absorbance increased slightly. Although, there are limitations of the use of a colorimetric assay to quantify silica ligands, the presence of free amines and thiols are undoubtedly located on the surface of the SIONPs.

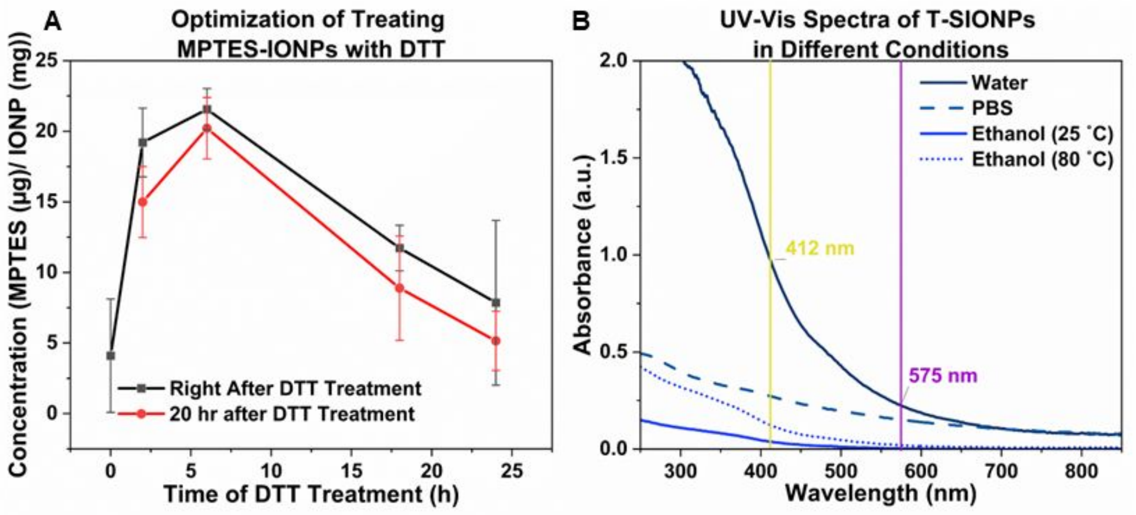

Figure S6. Scattering plot of the amount of MPTES found for 1 mg of MP-SIONPs reacted with DTT over different periods of time (h) right after reaction with DTT and 20 h after (A). UV-Vis spectra of T-SIONPs under nanopure water (solid black line), PBS (dashed black line), ethanol at room temperature (solid blue line), and ethanol at high temperature (dotted blue line) (B). The wavelength of interest (412 nm, yellow; 575 nm, purple) are shown.

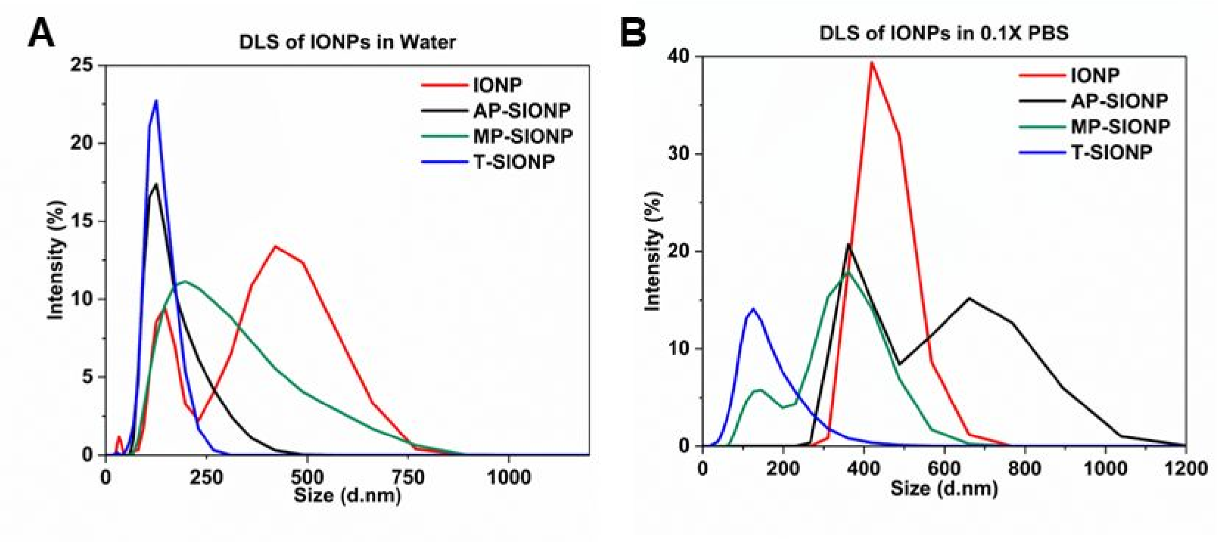

Figure S7. DLS results of IONPs (red) and AP-SIONPs (black), MP-SIONPs (green), T-SIONPs (blue) in water (A) and 0.1X PBS (B).

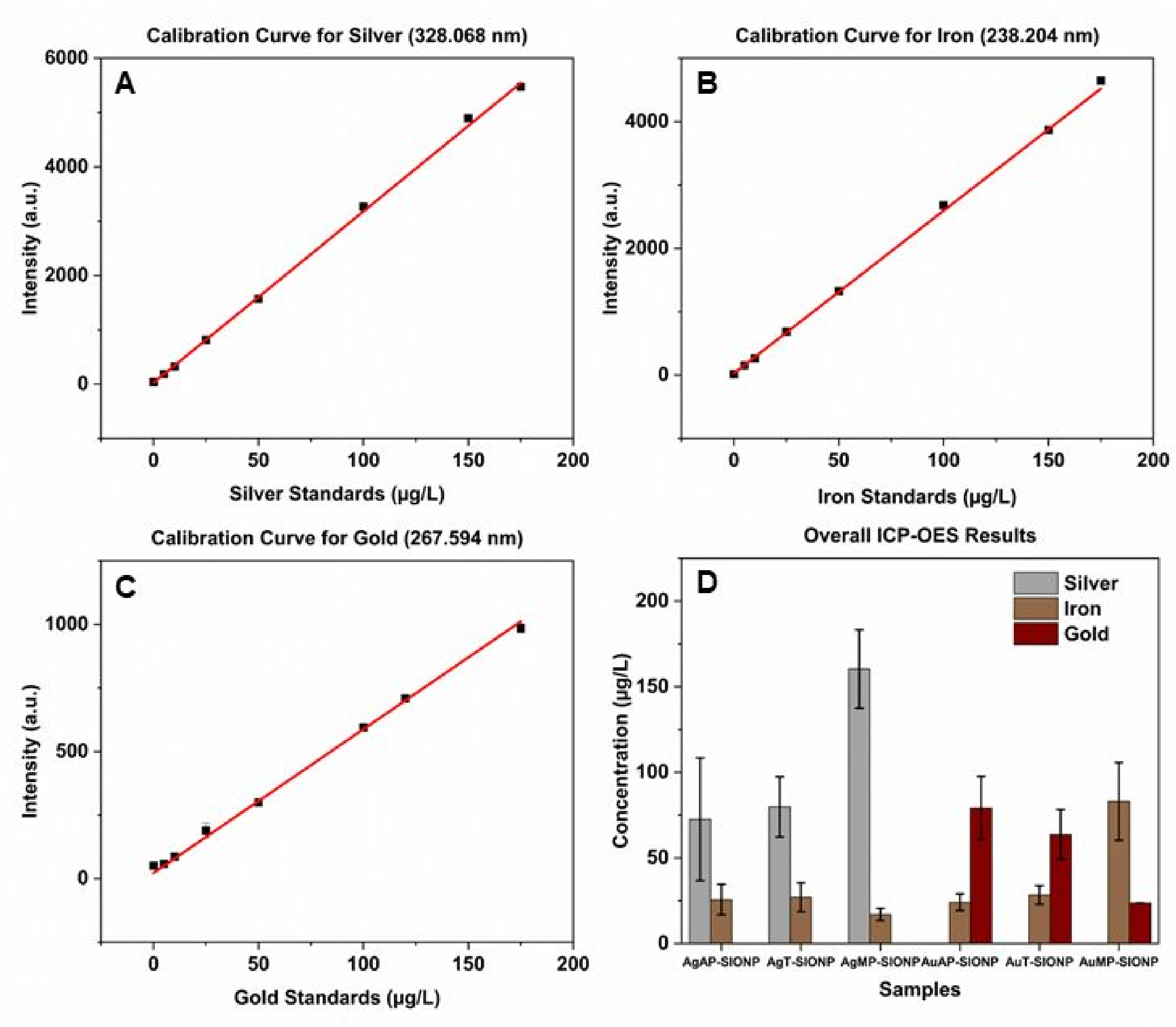

Figure S8. Calibration curves for silver (A), iron (B), and gold (C) after a series of standards for each element were prepared and analyzed with ICP-OES (R^2=0.99). The overall elemental concentration (µg/L) is compared for each of the six samples (AgAP-APTES, AgT-SIONP, AgMP-SIONP, AuAP-SIONP, AuT-SIONP, AuMP-SIONP).

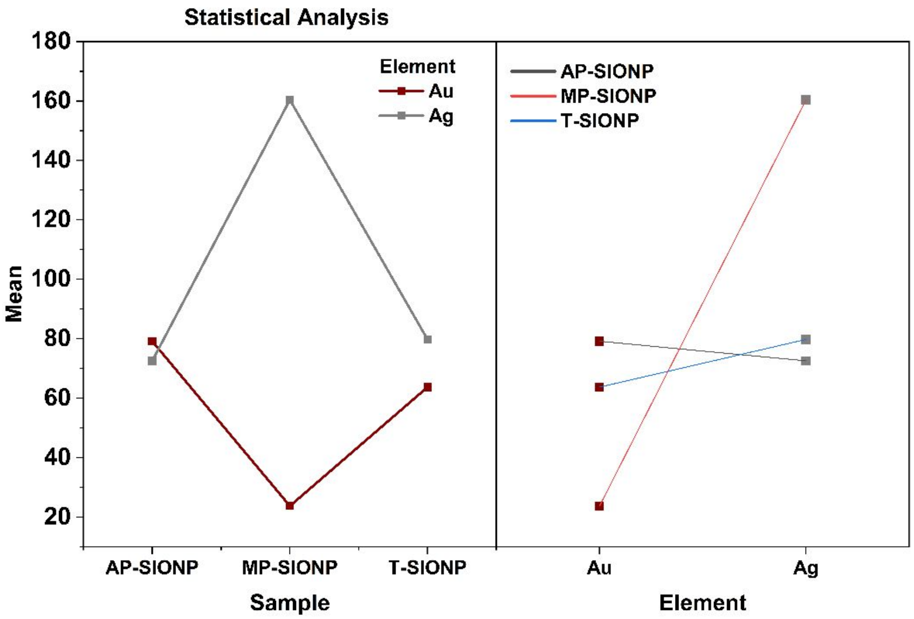

Figure S9. Scattering plots of the mean populations of the samples (AP-SIONP, MP-SIONP, and T-SIONP) and elements (Ag and Au) generated by two-way ANOVA.

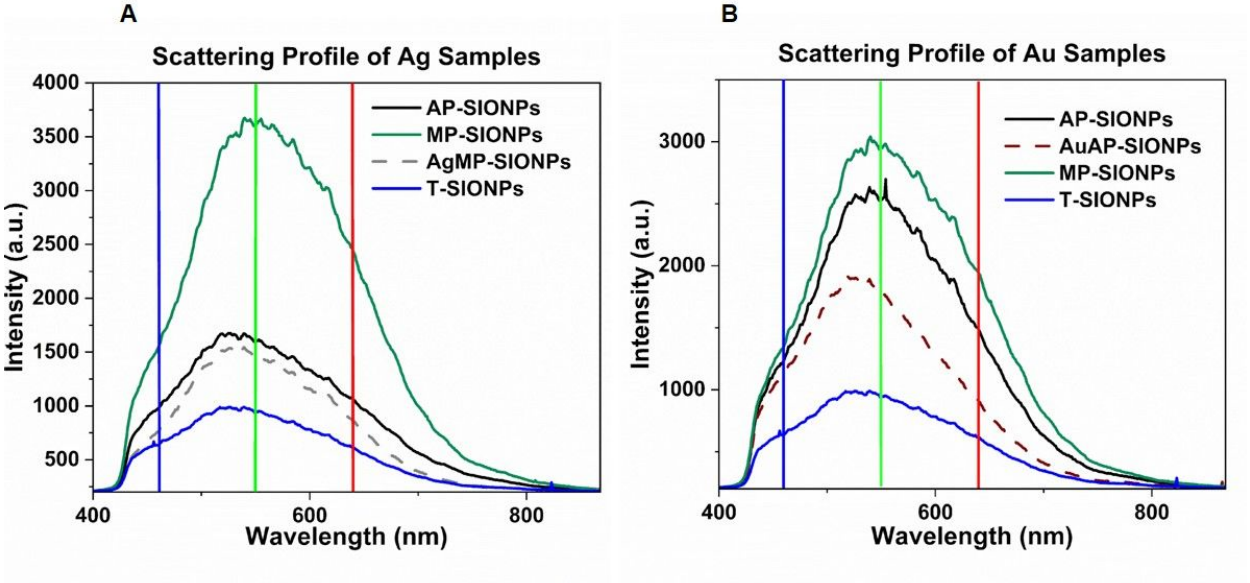

Figure S10. Scattering profile of AgNPs (grey dotted line), AgAP-SIONPs (black), AgMP-SIONP (green) and T-SIONP (blue) (A). Scattering profile of AuNPs (maroon dashed line), AuAP-SIONPs (black), AuMP-SIONP (green) and T-SIONP (blue) (B).

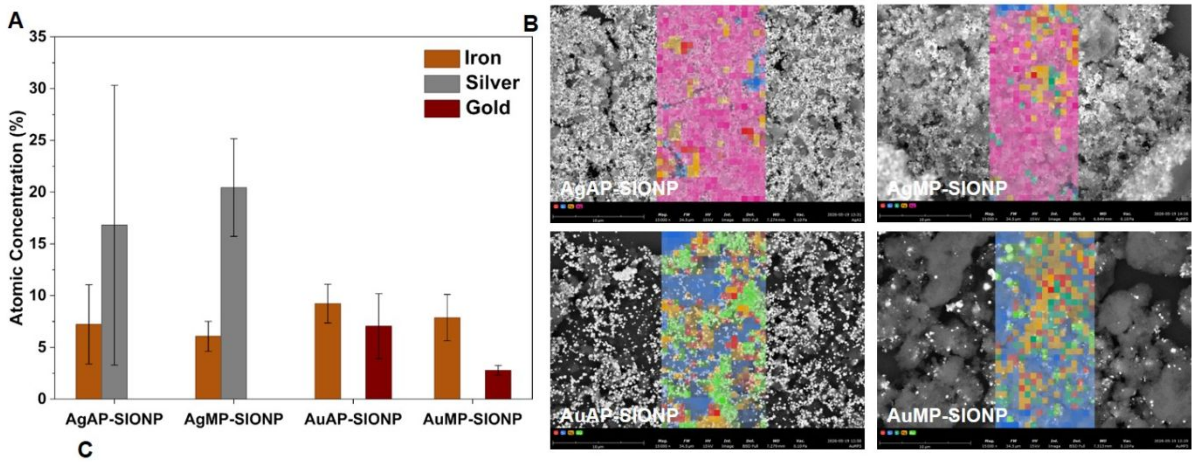

C

Sample	Fe	Ag	Au	O	Si	S
AgMP-SIONPs	6.1 ± 1.4	20 ± 5	____________	29 ± 5	41 ± 11	2.7 ± 0.8
AgAP-SIONPs	8.0 ± 4.7	17 ± 13	____________	26 ± 17	48 ± 5	____________
AuMP-SIONPs	7.8 ± 2.2	____________	2.8 ± 0.5	49 ± 8	49 ± 8	3.0 ± 0.7
AuAP-SIONPs	9.2 ± 1.9	____________	7.0 ± 3.1	40 ± 11	42 ± 15	____________

Figure S11. Graph depicting the atomic composition analysis for Ag, Au and Fe using EDS-SEM (n = 36) (A). Representative EDS-SEM images of the Ag- and Au-decorated nanomaterials (B). Atomic composition analysis using EDS-SEM (n = 36) (C). Note: N is not included because is notoriously challenging to detect with an EDS due to its peak being less than 0.4 kV and can easily overlap with other elements.

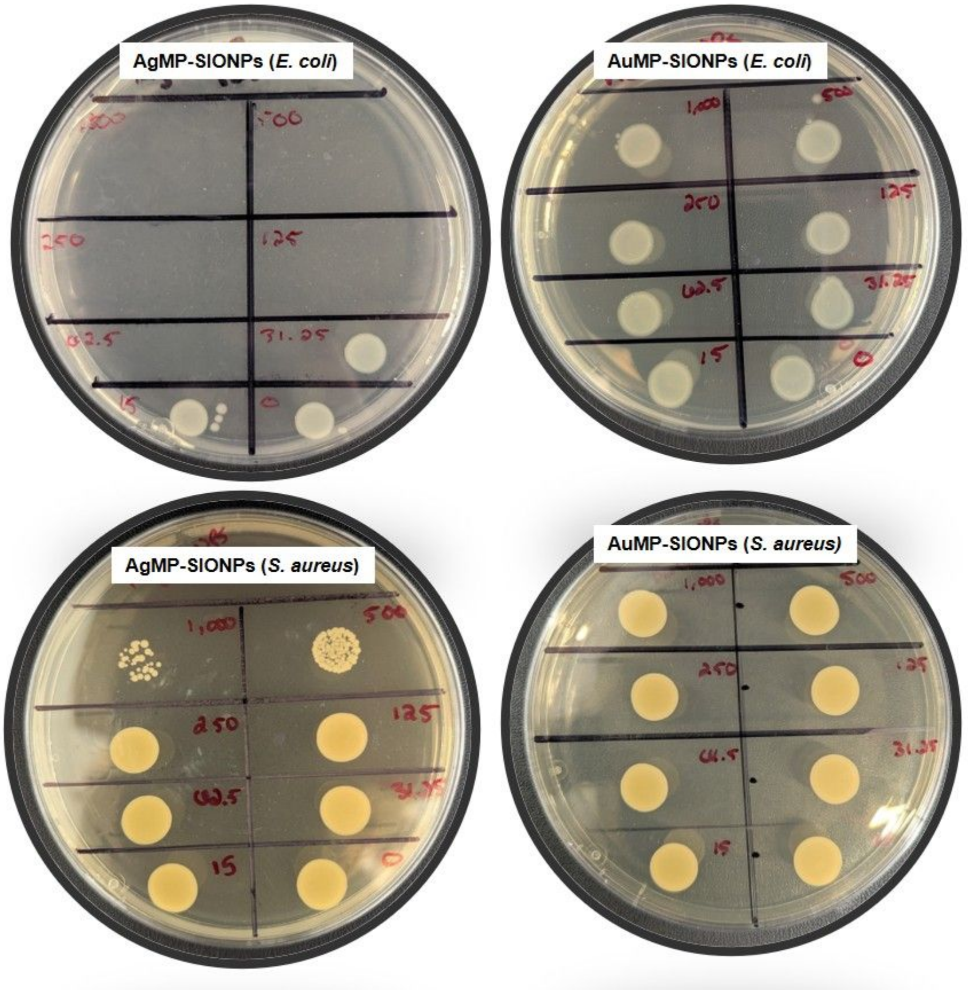

Figure S12. Images of agar plates after the supernatant post treatment was added. The supernatant of the optimal SIONPs is included (AgMP-SIONPs 1, 0.5, and 0.25 mg) and compared to AuAP-SIONP (1 mg) for comparison.
